# Supplementary figures and images for: Submicron Topographically Patterned 3D Substrates Enhance Directional Axon Outgrowth of Dorsal Root Ganglia Cultured Ex Vivo
Source: Biomolecules. 2022 Jul 30;12(8):1059. doi: 10.3390/biom12081059 (PMC9405616; doi:10.3390/biom12081059)

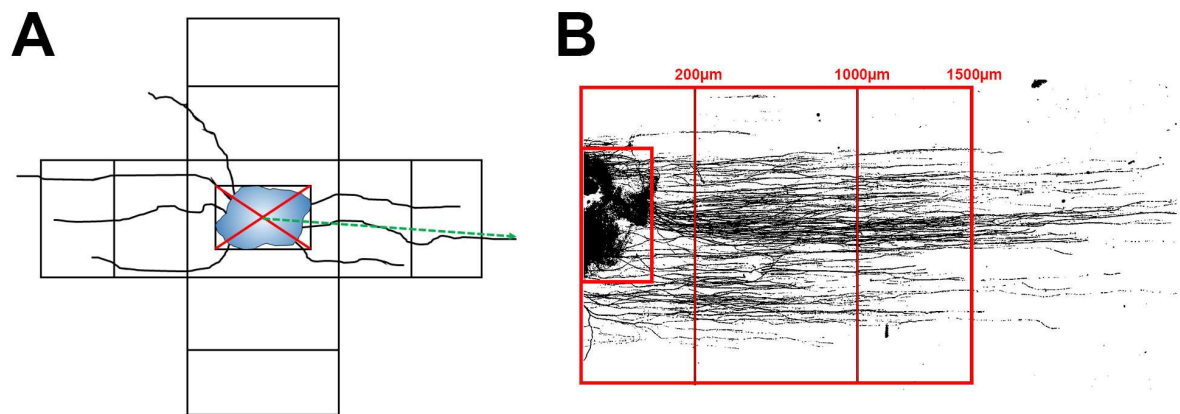

Figure S1: DRG axon measurement grids

Supplement: Supplementary file 1 [file biomolecules-12-01059-s001.zip › biomolecules-1775118-Figure S1.pdf]

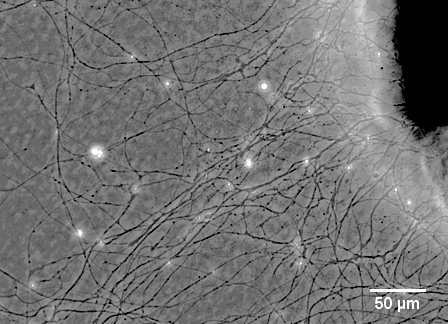

Supplement: Supplementary file 1 [file biomolecules-12-01059-s001.zip › Video S1 Axon regeneration on a flat surface.gif]

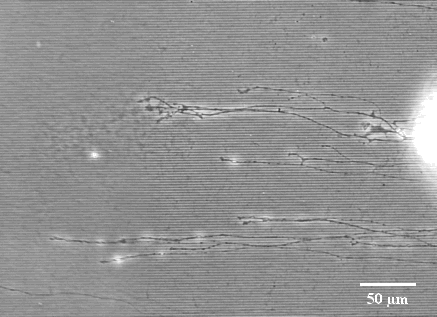

Supplement: Supplementary file 1 [file biomolecules-12-01059-s001.zip › Video S2 Axon regeneration on a 1400 nm surface.gif]
